# Pterygomaxillary space infection complicated by meningitis due to *Streptococcus constellatus*: Two case reports and literature review

**DOI:** 10.1097/MD.0000000000043468

**Published:** 2025-07-18

**Authors:** Baolin Jia, Ruihong Liu, Qiang Wang, Sen Yang

**Affiliations:** a Department of Oral and Maxillofacial Surgery, Suining Central Hospital, Suining, Sichuan Province, China.

**Keywords:** meningitis, multidisciplinary management, next-generation sequencing, odontogenic infection, pterygomaxillary space infection, *Streptococcus constellatus*, temporomandibular joint disorder

## Abstract

**Rationale:**

*Streptococcus constellatus* is a common oral bacterium implicated in pterygomaxillary space infections. Due to the deep anatomical location of these infections, they are often misdiagnosed as temporomandibular joint disorders. Although rare, concurrent meningitis in immunocompetent individuals can occur and may be life-threatening.

**Patient concerns:**

We report 2 older male patients who presented with facial pain and headache, initially misdiagnosed as temporomandibular joint disorders. Their conditions rapidly progressed to fever, altered mental status, and neurological deficits.

**Diagnoses:**

Cerebrospinal fluid analysis and metagenomic next-generation sequencing confirmed *S constellatus* meningitis. Computed tomography or magnetic resonance imaging revealed concurrent pterygomaxillary space infections.

**Interventions:**

Both the patients received targeted antibiotic therapy (meropenem followed by vancomycin/linezolid plus metronidazole); 1 patient also underwent surgical drainage.

**Outcomes:**

Both the patients recovered completely without neurological sequelae.

**Lessons:**

Although *S constellatus* meningitis is rare, odontogenic sources should be considered in patients with poor oral hygiene. Our report highlights the diagnostic value of next-generation sequencing for early pathogen detection in cerebrospinal fluid.

## 1. Introduction

Bacterial meningitis is a serious neurological condition with various potential sources of infection, including bacterial endocarditis, HIV infection, immunosuppressive therapy, and disruption of the protective blood–brain barrier following neurosurgery. Infections may also originate from otitis media or sinusitis or be secondary to other pathologies. However, the source of infection remains undetermined in 10% to 20% of cases.^[1]^ In these cases, odontogenic and maxillofacial infections are rare contributors. Other than *Streptococcus pneumoniae*, Streptococci rarely cause acute bacterial meningitis except in neonates. *S*treptococcus *constellatus* (*S constellatus*), a conditionally pathogenic oral bacterium, often causes maxillofacial interstitial infections and systemic septic infections in immunocompromised patients. However, *S constellatus*-induced meningitis is sporadic in immunocompetent adults. The specific type of Streptococcus is often related to the source of infection. This relationship could significantly influence the clinical presentation and prognosis.^[2]^ Therefore, understanding the characteristics of meningitis caused by different streptococcal species is essential for clinical diagnosis and treatment. This study reports 2 cases of pterygomaxillary space infection complicated by meningitis caused by *S constellatus*, initially misdiagnosed as temporomandibular joint disorder (TMD) and trigeminal neuralgia, which led to a delay in appropriate antibiotic therapy and progression of neurological symptoms.

## 2. Case reports

### 2.1. Case 1

**Figure 1.**
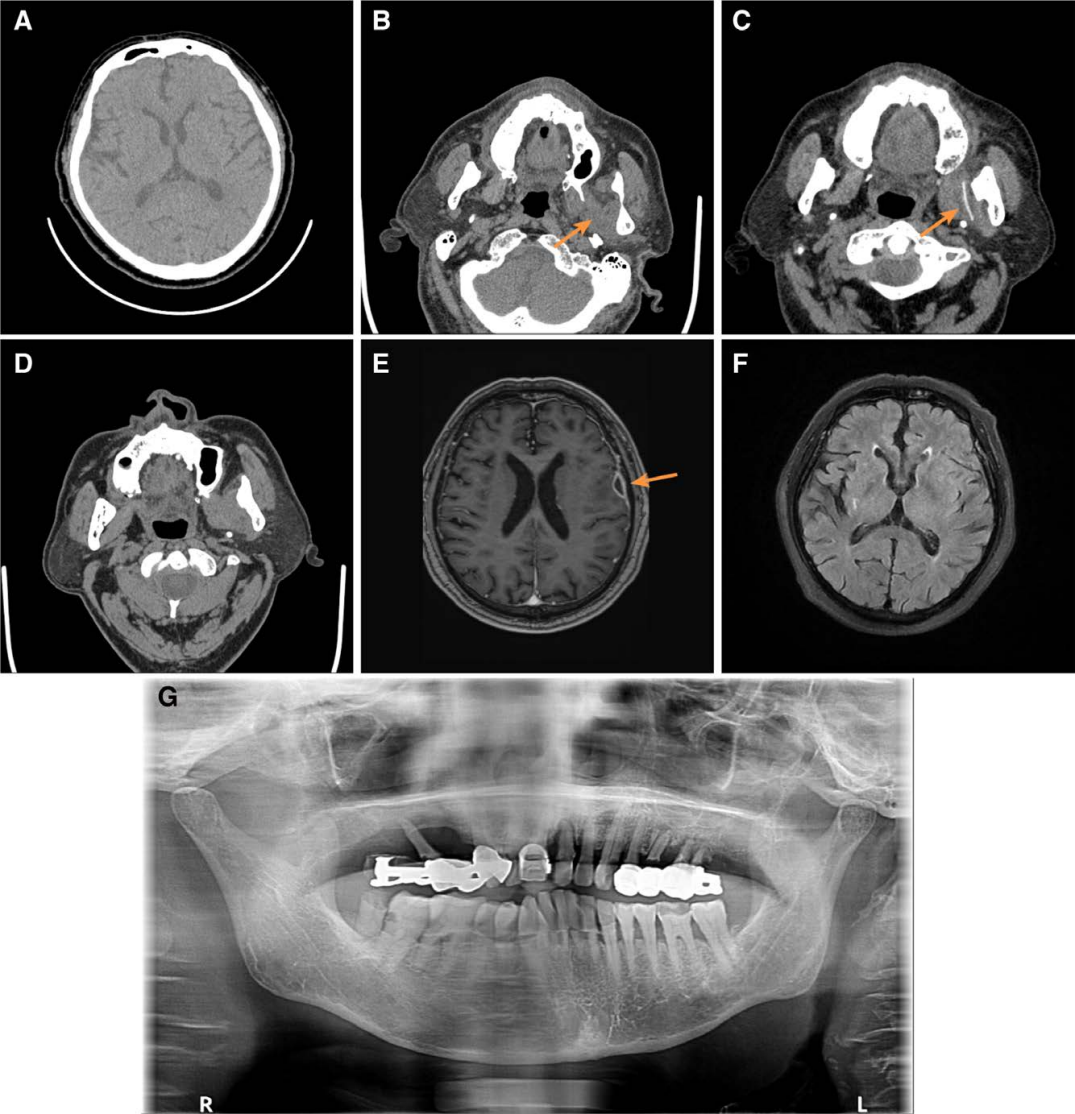
Imaging and clinical course of case 1. (A) Brain CT on admission. (B) Swelling of ectopterygoid muscle (arrow). (C) Incision and drainage with drain placement (arrow indicates drainage tube). (D) Review after drain removal. (E) Brain abscess formation (arrow). (F) Follow-up cranial MRI after 3 months. (G) Intraoral dental condition. CT = computed tomography.

A 70-year-old man presented to the Department of Stomatology at Suining Central Hospital 1 week earlier with pain during mouth opening, accompanied by a mild headache. He denied any history of infection elsewhere and had no long-term use of glucocorticoids or immunosuppressive drugs. The patient did not exhibit facial swelling or dysphagia but showed moderate limitations in mouth opening and pain in the preauricular region. The initial diagnosis was TMD, and symptomatic treatment was administered. However, his headache worsened. One week later, he developed a fever and increased difficulty with chewing, prompting a visit to the emergency department. He was lethargic, exhibited neck stiffness, and had a temperature of 38.1°C. Laboratory tests revealed a white blood cell count of 21.2 × 10^9^/L, a neutrophil count of 19.49 × 10^9^/L, an ultrasensitive C-reactive protein of 110.62 mg/L, and a procalcitonin of 2.22 ng/mL. Computed tomography (CT) angiography of the head and neck, as well as CT imaging of the skull and brain, showed no abnormalities (Fig. 1A). Bacterial meningitis was suspected and he was admitted to the Department of Neurology for further treatment.

Upon admission, meropenem (2 g every 8 hours) was initiated for anti-infection therapy. Cerebrospinal fluid (CSF) analysis revealed elevated white blood cells and protein levels, confirming bacterial meningitis. Although CSF cultures were negative, *S constellatus* and *Prevotella intermedia* were detected by cerebrospinal fluid next-generation sequencing (NGS) and blood cultures, leading to a final diagnosis of *S constellatus* meningitis. On the fifth day of hospitalization, the patient presented with reduced speech, unresponsiveness, and a fever of 38.5°C. Vancomycin (500 mg every 6 hours) was added based on recommendations from the infection and pharmacy departments. The modified anti-infective regimen effectively reduced inflammatory markers, and the patient’s body temperature returned to normal. Although his headache symptoms improved, facial pain during mouth opening and chewing persisted.

On the 13th day of hospitalization, a repeat facial CT revealed occult infection and swelling of the surrounding muscles in the left pterygomaxillary space (Fig. 1B). Aspiration yielded purulent fluid. The patient was transferred to the maxillofacial surgery department, where intraoral incision and drainage were performed (Fig. 1C) resulting in significant headache relief, although intraoral pus culture was negative. After 1 week of continued anti-infection therapy, the intraoral drainage tube was removed, and facial CT revealed no new infection (Fig. 1D). Cranial magnetic resonance imaging (MRI) showed a brain abscess of approximately 1.0 cm in diameter (Fig. 1E). Neurosurgery recommended conservative treatment since the lesion was <2.5 cm. Following discharge, the patient continued oral antibiotic therapy. One month later, he underwent periodontal treatment, and the focal intraoral tooth was extracted (Fig. 1G). Three months later, cranial MRI showed no signs of a brain abscess (Fig. 1F), and the patient exhibited no neurological sequelae, with a good recovery.

### 2.2. Case 2

**Figure 2.**
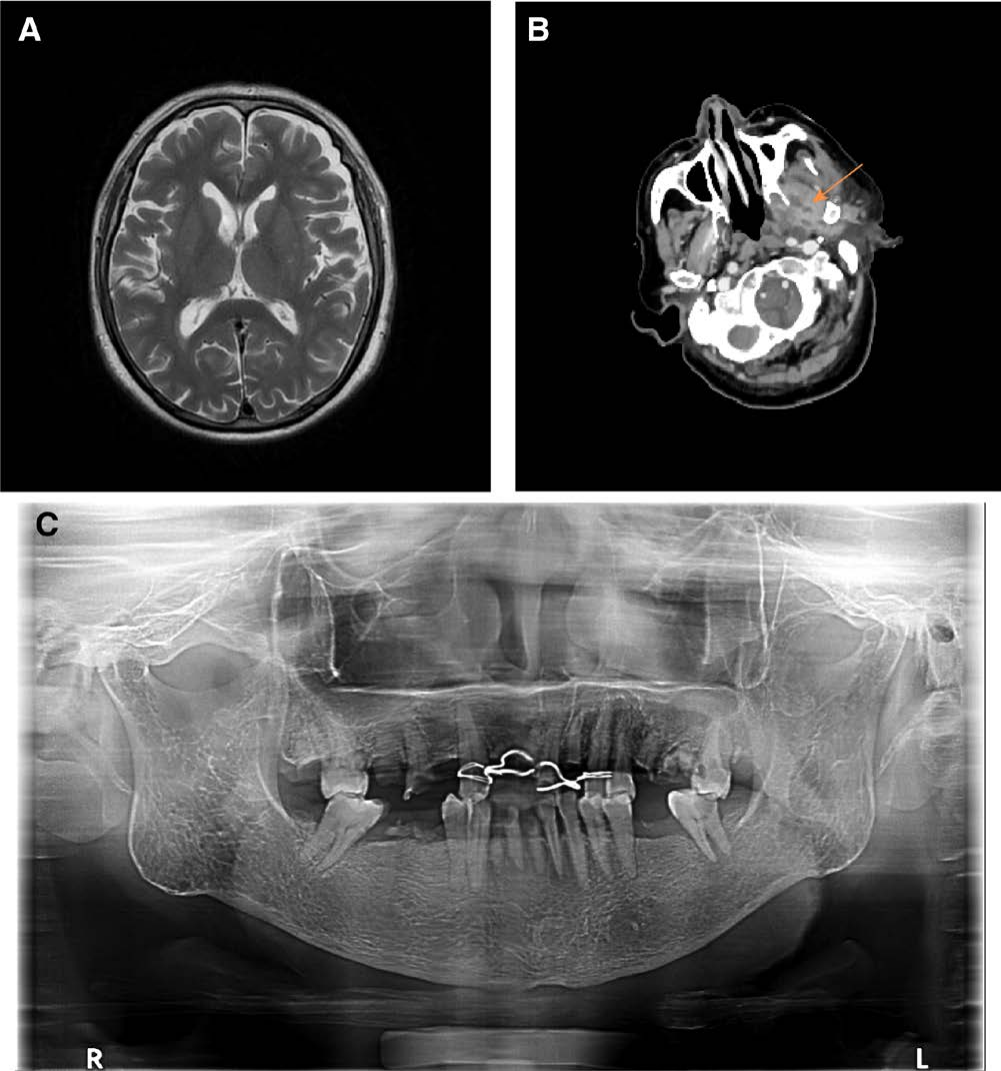
Imaging and clinical findings of case 2. (A) Cranial MRI on admission. (B) Extrapterygoid swelling with multiple small hypodense shadows (arrow). (C) Intraoral dental condition. MRI = magnetic resonance imaging.

A 76-year-old man, previously healthy, presented to the Departments of Dentistry and Neurology in January with left-sided facial pain. After an MRI of the trigeminal nerve, he was diagnosed with trigeminal neuralgia and temporomandibular arthritis and was treated symptomatically. However, 1 week later, the patient developed intermittent fever, facial swelling and neurological symptoms, including drowsiness and slurred speech. Laboratory tests revealed a white blood cell count of 20.8 × 10^9^/L, neutrophil count of 18.45 × 10^9^/L, ultrasensitive C-reactive protein of 144.17 mg/L, and calcitonin gene of 0.54 ng/mL. CSF analysis showed a leukocyte count of 7915 × 10^6^/L and protein quantification of 2.43 g/L. Cranial MRI and magnetic resonance angiography were unremarkable (Fig. 2A), but facial CT with contrast showed infection of the left pterygomaxillary space and swelling of the surrounding masticatory muscles (Fig. 2B). The patient was diagnosed with pterygomaxillary space infection complicated by meningitis and was admitted to the neurology department.

Upon admission, he was treated with meropenem (2 g every 8 hours) and dehydration therapy with mannitol and glycerol fructose. Despite treatment, his temperature rose to 39°C, and he became lethargic and uncooperative. A multidisciplinary consultation involving neurosurgery, infection specialists, pharmacy, maxillofacial surgery, and critical care was organized. On day 3, CSF mNGS detected a mixed infection of *Porphyromonas gingivalis*, *Porphyromonas deheparinum*, *Prevotella pleuropneumonia*, and *S constellatus*, with predominant anaerobic oral colonization. *S constellatus* was also isolated from blood cultures, and metronidazole was co-administered. After 1 week, his inflammatory markers decreased significantly, and facial swelling and pain improved, though intermittent high fever persisted. Linezolid was added based on the pharmacy department’s recommendation. After 5 days, his mental status improved, his fever resolved, and his headache symptoms significantly diminished. After 10 days of step-down therapy, his condition stabilized, and he was discharged. One month later, the suspected focal tooth was extracted in the dental clinic (Fig. 2C), and the patient received systemic periodontal treatment.

## 3. Discussion

Odontogenic meningitis is often difficult to diagnose. In the present cases, odontogenic bacteria were identified in the cerebrospinal fluid via NGS, and blood cultures yielded the same bacteria, alongside clinical and imaging evidence of pterygomaxillary space infection. Despite the absence of tooth extractions or recent dental procedures, both patients had severe periodontitis, and we hypothesize that the infection originated from the periodontium. Poor oral hygiene, compounded by bacterial virulence, leads to infection in the pterygomaxillary space. Through chewing, bacteria can breach the blood–brain barrier and cause meningitis. Rapid and accurate identification of the causative organisms is critical for improving patient prognosis. The traditional Gram stain is not sufficiently sensitive, particularly for anaerobic infections. NGS technology is increasingly utilized for the early diagnosis and precise treatment of infectious diseases.^[3]^ In this study, NGS helped identify *S constellatus*, clarifying the infection source and guiding the adjustment of anti-infective therapy.

*S*treptococcus *constellatus* is an anaerobic, Gram-positive bacterium commonly found in the oral cavity, nasal cavity, and pharynx. Its risk of infection increases when host immunity is compromised, or other underlying diseases are present. This pathogen can cause abscesses in the lungs, liver, and heart.^[4,5]^ In our clinical experience, it is often detected in severe maxillofacial interstitial infections. *S constellatus* produces various extracellular hydrolytic enzymes, likely explaining its ability to cause abscesses.^[6]^ Antibiotics should cover this pathogen, and it is important to recognize that odontogenic meningitis is typically polymicrobial.^[7]^ Therefore, early empirical treatment with broad-spectrum antibiotics, followed by targeted therapy based on drug susceptibility testing, is recommended.

Since the source of infection greatly influences clinical outcomes, it is essential to recognize each pathogen’s unique clinical characteristics and treatment strategies. In our review of 5 studies on odontogenic meningitis caused by *S constellatus* (Table 1), the patients were typically older with a history of underlying disease, cranial trauma, or periodontal infection. Most cases in the review received prolonged antibiotic therapy, 4 of them required a combination of antibiotics and surgery, and all had a favorable prognosis.^[8–12]^ Our 2 cases had no history of immunocompromised states or recent tooth extractions, but periodontal disease was identified as the source of infection based on mNGS and pathogen testing.

**Table 1:** Summary of cases of odontogenic meningitis caused by *S constellatus*.

Authors	Age	Sex	Underlying disease	Oral medical history	Initial symptoms	Treatment method	Anti-infective therapy duration	Prognosis
Pak S, et al^[8]^	60	Male	Hypertensive, atrial fibrillation	Tooth extraction	Epilepsy	Surgeries, Antibiotics	6 week	Survive
Chheda LV, et al^[9]^	54	Male	Diabetes	Tooth extraction	Vision loss	Antibiotics	12 week	Survive
Akashi, et al^[10]^	68	Male	History of cerebral infarction	Periodontitis	Memory impairment, disequilibrium	Surgeries, Antibiotics	6 week	Survive
Marques da Silva R, et al^[11]^	60	Female	Diabetes	Periodontitis	Reduced cognitive function	Surgeries, Antibiotics	2 week	Survive
Yoshizawa, Kunio, et al^[12]^	77	Male	COPD	Inflammation of tooth pulp	Tachycardia, decreased level of consciousness	Surgeries, Antibiotics	15 week	Survive

Odontogenic meningitis does not typically present with the symptoms of localized odontogenic infection. In these cases, the initial symptoms, such as pain upon mouth opening and mild headache, are often mistaken for TMD or trigeminal neuralgia. Without clear oral health information or medical history, misdiagnosis is common. However, key diagnostic clues emerge as the disease progresses, particularly with symptoms such as fever, altered mental status, and neck stiffness. Oral surgeons must pay close attention to a patient’s history of possible odontogenic infections, even when they do not present with acute oral symptoms. Maxillofacial infections, especially pterygomaxillary space infections, are frequently overlooked. These infections typically lack significant facial swelling and may only present as limited mouth opening or localized pain, often mistaken for TMDs or noninfectious conditions. Therefore, early imaging, particularly CT or MRI, is essential for detecting occult infections. There is no standardized guideline for treating intracranial infections of odontogenic origin, but multidisciplinary collaboration throughout the treatment process is crucial. In both cases, a multidisciplinary team of neurosurgery, dentistry, infection specialists, pharmacy, and critical care enabled rapid and precise treatment decisions, improving the treatment outcome.

In conclusion, *S constellatus* meningitis is a rare but serious complication caused by odontogenic infections. This case highlights the need for vigilance regarding deep infections occurring in anatomical spaces such as the pterygomaxillary space, which may serve as potential sources of central nervous system involvement. NGS can provide an effective adjunct to early pathogen identification when the diagnosis is difficult to confirm by traditional methods. Multidisciplinary collaboration and timely intervention are important for improving prognosis.

## Author contributions

**Data curation:** Baolin Jia.

**Resources:** Ruihong Liu.

**Supervision:** Qiang Wang.

**Visualization:** Baolin Jia.

**Writing – original draft:** Baolin Jia.

**Writing – review & editing:** Sen Yang.
